# Cognitive function following diabetic ketoacidosis in young children with type 1 diabetes

**DOI:** 10.1002/edm2.412

**Published:** 2023-02-14

**Authors:** Simona Ghetti, Nathan Kuppermann, Arleta Rewers, Sage R. Myers, Jeff E. Schunk, Michael J. Stoner, Aris Garro, Kimberly S. Quayle, Kathleen M. Brown, Jennifer L. Trainor, Leah Tzimenatos, Andrew D. DePiero, Julie K. McManemy, Lise E. Nigrovic, Maria Y. Kwok, Cody S. Olsen, T. Charles Casper, Nicole S. Glaser, Pediatric Emergency Care Applied Research Network (PECARN) DKA FLUID Study Group, Roger Lewis, Jeffrey Blumer, Andrew Bremer, Thomas Cook, Beth Slomine, Kathleen Meert, Jerry Zimmerman, Robert Hickey

**Affiliations:** ^1^ Department of Psychology University of California, Davis Davis California USA; ^2^ Center for Mind and Brain University of California, Davis Davis California USA; ^3^ Department of Emergency Medicine University of California Davis Health, University of California Davis, School of Medicine Davis California USA; ^4^ Department of Pediatrics University of California Davis Health, University of California Davis, School of Medicine Davis California USA; ^5^ Division of Emergency Medicine, Department of Pediatrics, The Colorado Children's Hospital University of Colorado‐Denver School of Medicine Aurora Colorado USA; ^6^ Division of Emergency Medicine, Department of Pediatrics, Children's Hospital of Philadelphia Perelman School of Medicine at the University of Pennsylvania Philadelphia Pennsylvania USA; ^7^ Department of Pediatrics University of Utah School of Medicine Salt Lake City Utah USA; ^8^ Division of Emergency Medicine, Department of Pediatrics, Nationwide Children's Hospital The Ohio State University School of Medicine Columbus Ohio USA; ^9^ Departments of Emergency Medicine and Pediatrics, Rhode Island Hospital The Warren Alpert Medical School of Brown University Providence Rhode Island USA; ^10^ Division of Emergency Medicine, Department of Pediatrics, St. Louis Children's Hospital Washington University School of Medicine in St. Louis St. Louis Missouri USA; ^11^ Division of Emergency Medicine, Department of Pediatrics, Children's National Medical Center The George Washington School of Medicine and Health Sciences Washington District of Columbia USA; ^12^ Division of Emergency Medicine, Department of Pediatrics, Ann and Robert H. Lurie Children's Hospital of Chicago Northwestern University Feinberg School of Medicine Chicago Illinois USA; ^13^ Division of Emergency Medicine, Nemours/A.I. DuPont Hospital for Children Sidney Kimmel Medical College at Thomas Jefferson University Philadelphia Pennsylvania USA; ^14^ Division of Emergency Medicine, Department of Pediatrics Texas Children's Hospital, Baylor College of Medicine Houston Texas USA; ^15^ Division of Emergency Medicine, Department of Pediatrics Boston Children's Hospital, Harvard Medical School Boston Massachusetts USA; ^16^ Division of Emergency Medicine, Department of Pediatrics, New York Presbyterian Morgan Stanley Children's Hospital Columbia University College of Physicians and Surgeons New York City New York USA

**Keywords:** cognitive function, diabetic ketoacidosis, early childhood, intelligence, type 1 diabetes

## Abstract

**Introduction:**

Young children with type 1 diabetes (T1D) may be at particularly high risk of cognitive decline following diabetic ketoacidosis (DKA). However, studies of cognitive functioning in T1D typically examine school‐age children. The goal of this study was to examine whether a single experience of DKA is associated with lower cognitive functioning in young children. We found that recently diagnosed 3‐ to 5‐year‐olds who experienced one DKA episode, regardless of its severity, exhibited lower IQ scores than those with no DKA exposure.

**Methods:**

We prospectively enrolled 46 3‐ to 5‐year‐old children, who presented with DKA at the onset of T1D, in a randomized multi‐site clinical trial evaluating intravenous fluid protocols for DKA treatment. DKA was moderate/severe in 22 children and mild in 24 children. Neurocognitive function was assessed once 2–6 months after the DKA episode. A comparison group of 27 children with T1D, but no DKA exposure, was also assessed. Patient groups were matched for age and T1D duration at the time of neurocognitive testing.

**Results:**

Children who experienced DKA, regardless of its severity, exhibited significantly lower IQ scores than children who did not experience DKA, *F*(2, 70) = 6.26, *p* = .003, partial *η* ^2^ = .15. This effect persisted after accounting for socioeconomic status and ethnicity.

**Conclusions:**

A single DKA episode is associated with lower IQ scores soon after exposure to DKA in young children.

## Graphical abstract

Young children with Type‐1 Diabetes may be at particularly high risk of cognitive decline following diabetic ketoacidosis (DKA), but studies of cognitive functioning in Type 1 diabetes typically examine school‐age children. The goal of the study was to examine whether a single experience DKA is associated with lower cognitive functioning in young children. We found that recently diagnosed 3‐to 5‐year‐olds who experienced one DKA episode, regardless of its severity, exhibited lower IQ than an age‐ matched group of patients with no DKA exposure.

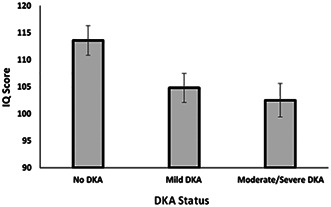

## 1 INTRODUCTION

Diabetic ketoacidosis (DKA), a common complication of type 1 diabetes (T1D),^1^ is associated with cognitive declines,^2^, ^3^, ^4^, ^5^, ^6^ even when no obvious neurological symptoms occur during DKA. Young children may be more vulnerable and at greater risk of developing these declines.^7^ Previous paediatric studies examining associations between DKA and cognition have focused on broad age ranges, extending beyond early childhood.^2^, ^3^, ^4^, ^5^, ^6^ In a small‐scale study, DKA was associated with larger memory declines if it occurred before age 7 years.^2^ However, children were assessed between 7 and 16 years of age, making it impossible to determine whether declines manifested soon after DKA exposure or emerged later and worsened over time.^7^ In a recent study from our group, lower pH at diagnosis of DKA, which was experienced at diabetes onset, was associated with lower intellectual quotient (IQ)^6^ in a sample of newly diagnosed 6‐ to 18‐year‐old children, suggesting small effects soon after the onset of T1D. However, there were no significant group differences in IQ scores between children who did and did not experience DKA; group differences were restricted to long‐term memory. In the current study, we asked whether group differences in IQ scores would be evident in young children tested 2–6 months after DKA at onset of T1D. Such a finding would suggest greater susceptibility to cognitive decline in young children.

## 2 METHODS

We recruited 3‐ to 5‐year‐old children who were newly diagnosed with T1D and exhibited DKA at onset from 12 centres participating in the Pediatric Emergency Care Applied Research Network (PECARN) Fluid Therapies Under Investigation in DKA (FLUID) trial.^8^ All of these children were randomized in the trial. We distinguished moderate/severe DKA (pH ≤ 7.19 or serum bicarbonate concentration ≤9 mmol/L) and mild DKA (pH between 7.20 and 7.25, or serum bicarbonate concentration between 10 and 15 mmol/L).^6^ Children with recent T1D diagnosis (<2 years), but no DKA episodes based on guardian history and medical review, were recruited from the paediatric diabetes clinics at the participating PECARN centres. Neurocognitive assessment sessions were delayed or rescheduled if children had either hypoglycaemia (glucose <70 mg/dL) or hyperglycaemia (glucose >350 mg/dL). All participants were English‐speaking.

We administered the English version of the Wechsler Preschool and Primary Scale of Intelligence (WPPSI‐III),^9^ which is validated for children ranging in age from 2 years and 6 months to 7 years and 3 months of age (normed IQ M = 100; SD = 15). Research coordinators trained by a study investigator with a doctoral degree in psychology administered the WPPSI. This measure was administered once between 2‐ to 6‐months after DKA for children with exposure to DKA or soon after recruitment for children with T1D without exposure to DKA. To limit the duration of the assessment and the child's fatigue, we only administered the core subtests of the WPPSI‐III corresponding to the minimal number of subsets that yielded a reliable and valid IQ score based on the test manual.^9^ The ability of the WPPSI to yield reliable and valid IQ scores from a subset of core subtests has been demonstrated across multiple versions.^9^, ^10^ For 3‐year‐olds, these core tests included four subtests, namely Block Design, Arithmetic, Vocabulary and Comprehension. For 4‐ and 5‐year‐olds, core tests included seven subtests, namely Block Design, Information, Matrix Reasoning, Vocabulary, Picture Concepts, Word Reasoning and Coding. The WPPSI is a highly regarded assessment tool to provide a comprehensive, reliable and valid measure of cognitive ability^9^ as demonstrated by its frequent used as an outcome measure in paediatric studies, including observational^11^, ^12^ and randomized clinical trial^8^, ^13^ investigations.

### 2.1 Statistical analyses

We tested for differences in frequency distribution of demographics as a function of DKA status (severe/moderate DKA vs mild DKA, vs. no DKA) using chi‐squared tests. We tested for differences in IQ scores as a function of DKA status using analysis of variance tests. We subsequently included socioeconomic status (SES), and ethnicity as covariates and conducted an analysis of covariance. Age was not included in the models because IQ scores are age‐normed. We performed the analyses using IBM SPSS Statistics (Version 27).

## 3 RESULTS

**FIGURE 1:**
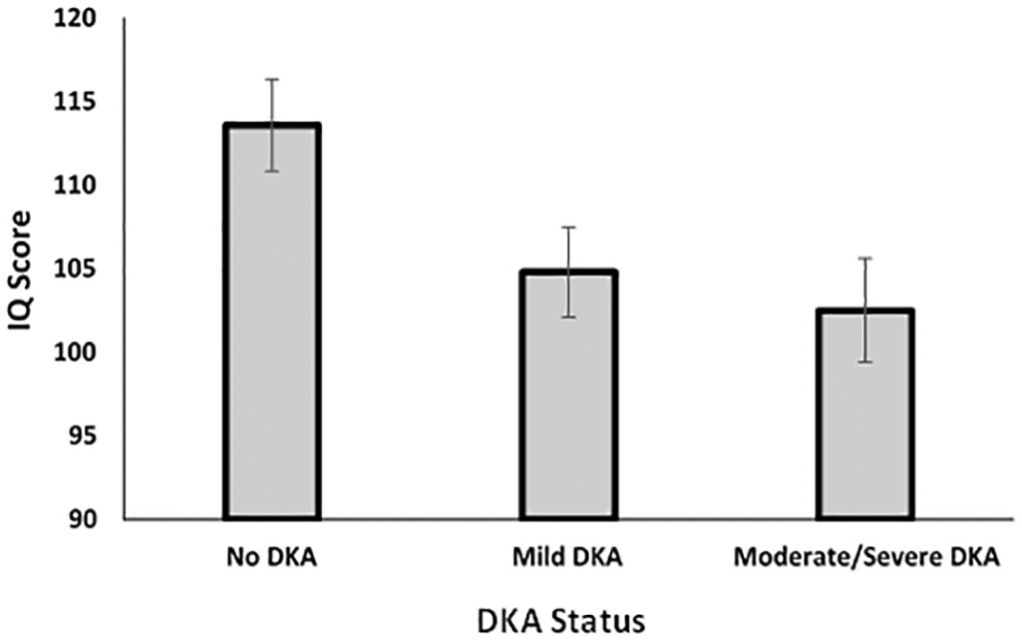
Intellectual quotient (IQ) score as a function of DKA status. Mean levels and standard errors around the means are reported. Children who did not experience DKA exhibited significantly higher IQ scores than children who experienced either mild or moderate/severe DKA (*p*s < .05). This analysis adjusts for SES and ethnicity.

We assessed 73 children (Table 1): 22 who had experienced moderate/severe DKA, 24 mild DKA and 27 no DKA. Patient demographic variables did not statistically differ as a function of DKA status (Table 1). However, children who experienced DKA, regardless of its severity, exhibited significantly lower IQ scores than children who did not experience DKA, *F*(2, 70) = 6.26, *p* = .003, partial *η* ^2^ = .15. Given nonsignificant trends for children who experienced DKA to be of lower SES or be of Hispanic ethnicity, we included SES and ethnicity as covariates in an additional analysis. The effect of DKA status persisted in this analysis, *F*(2, 63) = 3.88, *p* = .03, partial *η* ^2^ = .11 (Figure 1).

**TABLE 1:** Participant characteristics as a function of DKA status.

Characteristics	Non‐DKA (*N* = 27)	Mild DKA (*N* = 24)	Moderate/severe DKA (*N* = 22)	*p*‐Value*
Male: *N* (%)	16 (59.3)	11 (45.8)	9 (40.1)	.40
Age in years at testing: Mean (SD; Min–Max)	4.6 (0.8; 3.2–5.9)	4.6 (0.8; 3.1–5.8)	4.6 (0.9; 3.2–5.9)	.97
Age in years at T1D onset: Mean (SD; Min–Max)^a^	3.9 (0.9; 2–5)	3.8 (0.7; 2.9–5.5)	3.7 (1.1; 2.6–5.7)	.77
Glucose (mg/dL) at assessment: Mean (SD; Min–Max)^b^	250.7 (98.5; 74–464)	203.1 (79.1;73–350)	238.5 (112.1;72–485)	.22
Hypoglycaemic episodes prior to testing: *N* (%)	0 (0.0)	1 (4.2)	1 (4.5)	**
Socioeconomic Status: *N* (%)^c^				
High school/GED or less	2 (7.4)	4 (16.7)	3 (13.6)	.10
Some college/vocational school	5 (18.5)	7 (29.2)	10 (45.5)	
College degree or more	18 (66.7)	13 (54.1)	6 (27.3)	
Race: *N* (%)				
White	25 (92.6)	19 (79.2)	17 (77.3)	.48
Black	1 (3.7)	2 (8.3)	4(18.2)	
Other	1 (3.7)	3(12.5)	1 (4.5)	
Ethnicity: *N* (%)				
Hispanic	0 (0.0)	4 (16.7)	4 (18.2)	.07

^a^ Age at onset also corresponds to the age at which a single DKA episode was experienced in the Mild DKA and in the moderate/severe DKA groups.

^b^ Glucose levels correspond to the initial reading before testing. For the seven children who initially showed glucose ≥350, assessment was delayed until glucose was within the 70–350 range.

^c^ Maternal education (available only for 69 participants).

* *p*‐Values are from likelihood ratio chi‐square tests for categorical characteristics (those with *N* (%)), and analysis of variance *F*‐tests for continuous characteristics (those with mean (SD)).

** *p*‐Values were not calculated due to design‐imposed relationships with DKA Status.

## 4 DISCUSSION

Diabetic ketoacidosis has been associated with cognitive decline in children with T1D.^2^, ^3^, ^4^, ^5^ Our multi‐centre study provides new evidence of a strong and sizeable association between DKA status and IQ score in 3‐to 5‐year‐old children, just a few months after the onset of T1D. Our recruitment and assessment procedures were analogous to those used in a large study with 6‐ to 18‐year‐old children,^6^ in which no significant group differences in IQ scores were found between children with or without DKA exposure at diabetes onset, whose neurocgnitive function was also assessed 2‐ to 6‐ months after T1D diagnosis. Thus, following DKA, IQ declines may occur more quickly and/or be more robust in young children.

We recognize several limitations in the current study. First, the sample size is relatively small, although it is, to our knowledge, the largest study of cognitive functioning in very young children with T1D. Therefore, we were unable to measure the relative contribution of additional clinically relevant factors associated with cognitive functioning above and beyond DKA, such as acute kidney injury, which is a frequent complication of DKA.^14^ Second, we cannot exclude the possibility that the group differences in IQ scores reported here may depend, at least in part, on additional factors affecting the probability that children become exposed to DKA in the first place. For example, lower parental education may limit the ability to recognize early symptoms. Moreover, access to healthcare services is affected by such factors as SES and ethnicity. However, we controlled statistically for such factors in our analysis and the effects of DKA remained. Furthermore, previous findings have suggested that these socioeconomic factors play a sizeable role in previously diagnosed patients due to associations with the management of T1D.^6^ Future studies involving large, prospective samples are needed to fully characterize the relative contributions of these variables or other clinically relevant variables that may exacerbate cognitive declines in children with T1D (e.g. gestational age at birth^15^ and exposure to various environmental hazards^16^). Third, the absence of a comparison group of healthy children prevents us from examining associations with other risk factors for cognition in T1D.^17^ However, to the extent that the main focus of the research is the association with DKA, a comparison group of children with T1D without DKA is generally more informative.

## 5 CONCLUSIONS

The present study revealed that a single episode of DKA was associated with lower cognitive functioning in young children with T1D. These results suggest that young children may be particularly vulnerable to the negative effects of DKA, underscoring the importance of early detection of diabetes and prevention of DKA in young children.

## AUTHOR CONTRIBUTIONS

**Simona Ghetti:** Conceptualization (lead); data curation (lead); formal analysis (lead); funding acquisition (supporting); investigation (supporting); methodology (supporting); project administration (supporting)  writing – original draft (lead). **Nathan Kuppermann:** Conceptualization (lead); data curation (lead); formal analysis (supporting); funding acquisition (lead); investigation (lead); methodology (lead); project administration (lead); writing – original draft (supporting). **Arleta Rewers:** Data curation (supporting); investigation (supporting); project administration (supporting); supervision (equal); writing – review and editing (supporting). **Sage R. Myers:** Data curation (supporting); methodology (supporting); project administration (supporting); supervision (supporting); writing – review and editing (supporting). **Jeff E. Schunk:** Data curation (supporting); methodology (supporting); project administration (supporting); supervision (supporting); writing – review and editing (supporting). **Michael J. Stoner:** Methodology (supporting); project administration (supporting); supervision (supporting); writing – review and editing (supporting). **Kimberly S. Quayle:** Investigation (supporting); methodology (supporting); supervision (supporting); writing – review and editing (supporting). **Kathleen M. Brown:** Methodology (supporting); project administration (supporting); supervision (supporting); writing – review and editing (supporting). **Jennifer L. Trainor:** Investigation (supporting); methodology (supporting); supervision (supporting); writing – review and editing (supporting). **Leah Tzimenatos:** Investigation (supporting); methodology (supporting); supervision (supporting); writing – review and editing (equal). **Andrew D. DePiero:** Investigation (supporting); methodology (supporting); supervision (supporting); writing – review and editing (supporting). **Julie K. McManemy:** Methodology (supporting); project administration (supporting); supervision (supporting); writing – review and editing (supporting). **Lise E. Nigrovic:** Methodology (supporting); project administration (supporting); supervision (supporting); writing – review and editing (supporting). **Maria Y. Kwok:** Methodology (supporting); project administration (supporting); supervision (supporting); writing – review and editing (supporting). **Cody S. Olsen:** Formal analysis (supporting); validation (supporting); visualization (supporting); writing – review and editing (supporting). **T. Charles Casper:** Formal analysis (supporting); visualization (supporting); writing – review and editing (supporting). **Nicole S. Glaser:** Conceptualization (lead); data curation (lead); formal analysis (supporting); funding acquisition (lead); investigation (lead); methodology (lead); project administration (lead); writing – original draft (supporting).

## FUNDING INFORMATION

This study was supported by grant U01HD062417 from the Eunice Kennedy Shriver National Institute of Child Health & Human Development. This project was also supported in part by the Health Resources and Services Administration (HRSA), Maternal and Child Health Bureau (MCHB), Emergency Medical Services for Children (EMSC) Network Development Demonstration Program under cooperative agreement number U03MC00008, U03MC00001, U03MC00003, U03MC00006, U03MC00007, U03MC22684 and U03MC22685. This information or content and conclusions are those of the authors and should not be construed as the official position or policy of, nor should any endorsements be inferred by HRSA, HHS or the US Government. The funding agencies had no role in the study design and conduct.

## CONFLICT OF INTEREST STATEMENT

None of the authors have any financial arrangements that represent conflicts of interest related to the study.

## Data Availability

The data that support the findings of this study are available from the corresponding author upon reasonable request.
